# CytoNorm: A Normalization Algorithm for Cytometry Data

**DOI:** 10.1002/cyto.a.23904

**Published:** 2019-10-21

**Authors:** Sofie Van Gassen, Brice Gaudilliere, Martin S. Angst, Yvan Saeys, Nima Aghaeepour

**Affiliations:** ^1^ Department of Applied Mathematics Computer Science and Statistics, Ghent University Ghent Belgium; ^2^ Data Mining and Modeling for Biomedicine VIB Center for Inflammation Research Ghent Belgium; ^3^ Department of Anesthesiology, Perioperative and Pain Medicine Stanford University School of Medicine Stanford California; ^4^ Department of Biomedical Data Sciences Stanford University School of Medicine Stanford California

**Keywords:** normalization, mass cytometry, computational cytometry, barcoding

## Abstract

High‐dimensional flow cytometry has matured to a level that enables deep phenotyping of cellular systems at a clinical scale. The resulting high‐content data sets allow characterizing the human immune system at unprecedented single cell resolution. However, the results are highly dependent on sample preparation and measurements might drift over time. While various controls exist for assessment and improvement of data quality in a single sample, the challenges of cross‐sample normalization attempts have been limited to aligning marker distributions across subjects. These approaches, inspired by bulk genomics and proteomics assays, ignore the single‐cell nature of the data and risk the removal of biologically relevant signals. This work proposes CytoNorm, a normalization algorithm to ensure internal consistency between clinical samples based on shared controls across various study batches. Data from the shared controls is used to learn the appropriate transformations for each batch (e.g., each analysis day). Importantly, some sources of technical variation are strongly influenced by the amount of protein expressed on specific cell types, requiring several population‐specific transformations to normalize cells from a heterogeneous sample. To address this, our approach first identifies the overall cellular distribution using a clustering step, and calculates subset‐specific transformations on the control samples by computing their quantile distributions and aligning them with splines. These transformations are then applied to all other clinical samples in the batch to remove the batch‐specific variations. We evaluated the algorithm on a customized data set with two shared controls across batches. One control sample was used for calculation of the normalization transformations and the second control was used as a blinded test set and evaluated with Earth Mover's distance. Additional results are provided using two real‐world clinical data sets. Overall, our method compared favorably to standard normalization procedures. The algorithm is implemented in the R package “CytoNorm” and available via the following link: http://www.github.com/saeyslab/CytoNorm © 2019 The Authors. *Cytometry Part A* published by Wiley Periodicals, Inc. on behalf of International Society for Advancement of Cytometry.

High‐dimensional flow cytometry technologies, such as mass cytometry, are increasingly employed in large clinical studies to better understand the biological mechanisms of diseases 1, 2, 3. However, when samples are measured at multiple timepoints, technical variances between experimental batches can influence the measurements, resulting in so‐called batch effects. Technical variance can have various causes, including differences in sample collection time, stimulation, freezing, thawing, staining or instrument‐dependent effects.

One of the known instrument‐dependent issues specific to mass cytometry is signal fluctuation over time, due to changes in instrument performance. This signal drift is typically corrected by using polystyrene beads embedded with metals of known concentration 4. However, these beads will not capture differences due to experimental variability in sample preparation or changes in channels that are not represented by the bead signals. Therefore, additional normalization is still needed.

One solution to reduce batch effects is cellular barcoding 5, 6, an approach in which each individual sample is first stained with a specific set of tags, allowing unique identification of the samples. The samples can then be merged together and further processed as one, significantly reducing the experimental variability in sample handling, antibody staining and instrument detector sensitivity. However, the number of samples that can be uniquely labeled is limited (typically 20 or 96 samples), and clinical data sets may include more samples than a single barcode scheme can accommodate. Additionally, not all samples are always available at the same time, which complicates the barcoding process. Therefore, samples are often split in multiple groups, each of which are processed in one barcoded plate. While in this scenario, experimental variability is limited between samples on the same barcoded plate, separate plates suffer from similar experimental variability as two nonbarcoded samples would.

While several additional protocol standardization efforts are taking place 7, slight differences in sample handling between multiple batches are unavoidable 8. Computational techniques to remove this variance postmeasurement can offer a solution in those cases. A number of techniques have been proposed to align the distribution of markers across samples 9, 10, 11. However, these methods will align the distribution of each of the individual samples without making use of reference controls, which will also remove potentially biologically relevant differences in the distribution. In this work, we propose a pipeline for identification and normalization of technical variations using shared controls across multiple batches. Using control samples allows us to guarantee that the detected changes are only due to technical issues. Importantly, here we demonstrate that technical sources of variation can impact cell types differently, as was also described in Reference 10. They provided the option to normalize one marker at the time during the manual gating process, allowing the user to choose for which subpopulation the normalization is applied. In contrast, to allow a fully automated procedure, our algorithm first uses a clustering algorithm for automated cell type identification prior to normalization.

## Materials and Methods

### Mass Cytometry Data Sets

The main data set used in this study is a previously published mass cytometry data set immunoprofiling women during pregnancy 12. This data set included whole blood samples at four timepoints for 17 patients in the original cohort and an additional 10 patients in the validation cohort. Each patient was measured on a separately barcoded plate (including all timepoints and multiple stimulations (not used in this study)). In addition to the actual patient samples, we also made use of control samples. In the original cohort, blood from one healthy donor was taken along on every plate, both unstimulated and stimulated with Interferonα (IFNα) and Lipopolysaccharide (LPS). In the validation cohort, an additional healthy donor was taken along across all plates, to be used as a validation sample for our normalization protocol (four healthy donor samples per plate). The mass cytometry cytometry panel included 23 surface markers for cell phenotyping and 10 markers for functional analysis of the signaling responses (Supporting Information Table S1).

### Proposed Method

**Figure 1:**
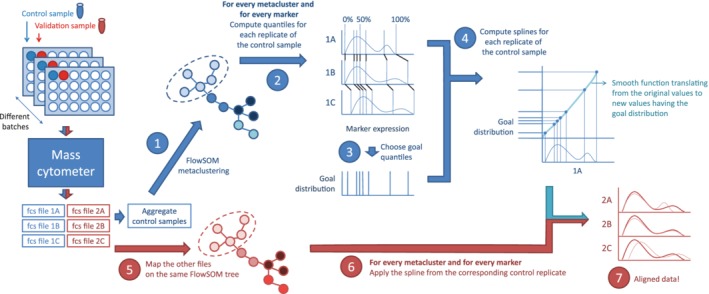
Schematic overview of the proposed workflow. First the batch effects are learned on the control sample (blue part). Afterward, the other samples can be normalized (red part). When a validation sample is included on the plates, we can evaluate whether these aliquot measurements become more similar as well, as they should after correct normalization. [Color figure can be viewed at http://wileyonlinelibrary.com]

A schematic overview of the proposed method is given in Figure 1. An identical control sample taken at one timepoint from one healthy control is included in each of the batches. Using the information from this control sample, our algorithm corrects for batch‐to‐batch variability, which might result from sample thawing, processing, staining and instrument particularities. The algorithm consists of two main parts: The batch effects are first modeled using the control sample, and afterward all samples can be normalized using the resulting models. This results in normalized data that is comparable between plates and can be used for further analysis.

#### Modeling the Batch Effects

To model the batch effects, we propose a four‐step pipeline (shown in blue in Fig. 1). First, it clusters the cells of the control samples to capture the different cell types present in the data. Next the quantiles are computed to capture the distribution of the cells over the marker expression values. A goal distribution is then determined based on the means of the quantiles. Finally monotone Hermite splines are computed to translate the original values to new values that follow the goal distribution.

We used FlowSOM for the automated cell population identification 13, Step 1 in Figure 1. The FlowSOM algorithm uses a two‐step clustering: The data are first divided in many more clusters than expected cell populations using a self‐organizing map (SOM) and next the resulting cluster centers are grouped using a consensus hierarchical clustering, resulting in the final cluster labels. By using this two‐step clustering, the FlowSOM algorithm can detect populations of varying sizes and shapes, without the computational overhead of density‐based clustering algorithms 14. To identify the populations from all batches, the training samples were first aggregated and the FlowSOM algorithm was applied to the aggregated sample. To limit the running time, a random subset was selected from each sample to use for training (1 million cells in total). All 37 markers were used for FlowSOM clustering. Once the clusters were determined, all original cells were mapped to their closest SOM‐cluster centers for further analysis.

By applying clustering on the original data first, we made the assumption that while the measurements might have shifted between the different samples, the differences between the cell types are still bigger than the shifts caused by the batch effects. If this is the case, the percentage of cells assigned to each of the clusters should be similar across all control samples. We evaluated this by computing the percentage of cells assigned to each of the clusters for each of the samples, and computing the coefficient of variation for each of the clusters. In the training set, this resulted in a maximal CV of 0.87 for 25 clusters. Once CV values higher than two occur (which was the case for 30 or more clusters), it is likely that some of the clusters are severely impacted by the batch effects, which renders the clustering step inappropriate as a preprocessing step before quantile normalization.

Once the FlowSOM algorithm was run, we applied the normalization per cluster. By processing only one cluster at a time, we assumed no further dependencies between the markers and normalized each marker separately. To describe the distribution of the cells for a specific marker, we made use of quantiles. A quantile indicates a boundary value for which a certain percentage of cells is lower, for example the 0.5 quantile corresponds to the median value, whereas the 0 quantile corresponds to the minimum value and the 1 quantile to the maximum. We computed 101 quantiles, from 0 to 1 in steps of 0.01, and obtained an overview of the distributions for each control sample per marker, per cluster (Step 2).

Once the distributions were determined for all control samples, a goal distribution can either be computed as the mean value for each of the quantiles over the different control samples (Step 3), or the result of one of the specific batches can be picked as the goal distribution.

To model the transformation needed to go from the actual distribution to the goal distribution, we used monotone hermite spline functions (as implemented in the monoH.FC method of the stats::splinefun function in R). A spline is a piece‐wise defined function, used to interpolate between given points, while still keeping a certain smoothness at the transitions between the piece‐wise defined functions. For each control sample, we used the original quantiles as the x‐values and the corresponding quantiles from the goal distribution as the y‐values to define the interpolation points. To avoid spurious extrapolation, we also added (0,0) and (8,8) to the interpolation points. The resulting spline function was then used to translate all original marker values to the new values, causing the data to become close to the goal distribution (Step 4).

For every control sample, this resulted in one spline for each pair of clusters and markers.

#### Normalizing the Data

The precalculated FlowSOM model and the splines were used to normalize the rest of the samples, as shown in red in Figure 1. First the new samples were mapped on the given FlowSOM clustering, to assign every cell to a cluster (Step 5). Then, for every measured value, the corresponding spline function (given the cluster, the marker and the control sample of relevance) was applied (Step 6). This way, we adapted the cell expression values in the new samples to reverse the shifts detected in the control samples. The variation left could then be assumed to only represent the real biological variation (which should be minimal for the second control sample).

### Alternative methods

We compared our proposed method with a few variations. Instead of using the 101 quantiles, we also tried an approach using only two quantiles, aligning either the minimum and maximum or the 0.001 and 0.999 quantiles. If only two quantiles are used, a linear function is modelled instead of using a spline function. Additionally, we also tried these methods either with or without the clustering step. In all these cases, we learned the models on the control samples, and afterward applied them on the validation samples.

$$x_{\mathrm{norm}}=\left(x\;–\;x_{q0.001}\right)/\left(x_{q0.999}\;–\;x_{q0.001}\right)$$

Next to these variations, which have been used in microarray normalization before 15, 16, we also compared our algorithm to GaussNorm 9, a technique previously proposed for the normalization of cytometry data. This algorithm does not allow to learn on control samples and translate the shifts to other samples afterward, so this was applied immediately on the set of sample of interest. It takes as input the expected number of peaks for every marker, which was manually determined by looking at the density plots. To enable application of a static gating build on one sample, we computed the landmarks used for alignment on one sample, and gave those landmarks as input for the normalization of all other samples.

### Evaluation

A first evaluation of CytoNorm was applied on the validation samples included in the second cohort of the pregnancy study. We used the control samples of the first healthy donor to train a model and then normalized the validation samples from the second healthy donor. We tested whether known populations (as defined by a manually adapted gating) aligned better after normalization. To quantify this alignment, we made use of the Earth Movers Distance (EMD,^17^). EMD is a distance measure specifically developed to compare distributions. To describe the distributions, we binned the data in bins of size 0.1 for every marker (on transformed data). For every manually gated cell population *p* and every marker *m*, we computed the pairwise EMDs across all the batches and took the maximum value. This indicates the maximum distances between two plates occurring in this data set. The lower this value is, the better. To evaluate how these distances change after normalization, we compute the EMDs for both the original data set and the normalized data sets. This allows us to compute the reduction in EMD, the percentage of the original distance that is eliminated by the normalization. To capture all this information in one number, we did not take into account the population‐marker pairs where both the original and normalized EMD values where lower than 2 (thus not impacted by the batch effects or the normalization) and compute the average over all other population‐marker pairs as a final score.

$${\mathrm{EMD}}_{p,m}=\max_{i,j\in \text{validation samples}}\mathrm{EMD}\left({\text{data}}_{i}\left[p,m\right],{\text{data}}_{j}\left[p,m\right]\right)$$

$${\text{Reduction}}_{p,m}=\frac{\text{original}\;{\mathrm{EMD}}_{p,m}-\text{normalized}\;{\mathrm{EMD}}_{p,m}}{\text{original}\;{\mathrm{EMD}}_{p,m}}$$

$$\text{Reduction}=\underset{\begin{array}{c} p\in \text{populations}\\ m\in \text{markers}\\ {\mathrm{EMD}}_{p,m}>2 \end{array}}{\text{mean}}{\text{Reduction}}_{p,m}$$

Additionally, we evaluate the normalization procedure on the patient samples of the pregnancy study. For this purpose, we make use of a manual gating defined on one control sample, and apply this as a static gating on all files. In contrast, we also have the population frequencies of the original publication, where all gates were adapted as needed on the individual files. We show that on a normalized data set, time and effort can be saved by getting relevant results without having to manually adapt all gates.

### Availability

This proposed algorithm is implemented in the R package “CytoNorm” and available on github at http://www.github.com/saeyslab/CytoNorm. As input, the user needs to provide the fcs files from the control samples, the fcs files that need to be normalized and labels indicating the batch origin for each file. Optionally, parameter settings for the FlowSOM algorithm and the number of quantiles can be chosen. In the end, a new set of fcs files is generated with normalized values. The pipeline used to generate the results described in this manuscript is available at http://www.github.com/saeyslab/CytoNorm_Figures. The fcs files and manual gating from the control samples from the original pregnancy cohort are available at flow repository ID FR‐FCM‐Z246. The fcs files and manual gatings from the validation pregnancy cohort are available at flow repository ID FR‐FCM‐Z247.

## Results

### Batch Effects Are Nonlinear and Can Be Cell‐Type Specific

**Figure 2:**
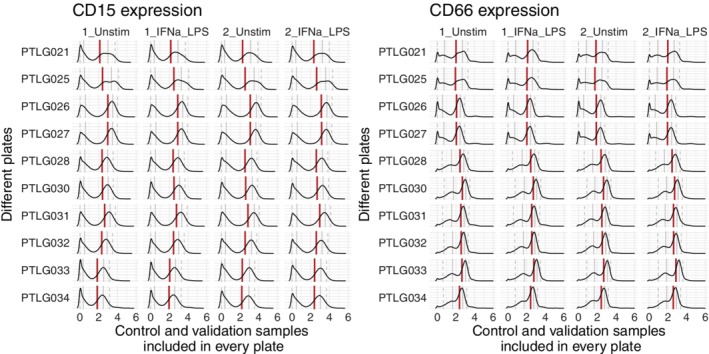
Overview of CD15 and CD66 expression of four samples, replicated across 10 plates (ordered by time of measurement). It is notable that all samples from the same plate had similar shifts in comparison to the other plates, which indicates that these shifts were caused by batch effects. The density plots show the arcsinh‐transformed expression values (cofactor 5). The red line represents the median value, the full grey lines represent the 0.25 and 0.75 quantiles and the dashed gray lines represent the 0.05 and 0.95 quantiles. For CD15 the negative peak (in this case represented by the 0.05 and 0.25 quantiles) was quite stable, but the positive peak shifted between the plates. For CD66, especially the negative peak was impacted, although the positive peak also underwent slight shifts. [Color figure can be viewed at http://wileyonlinelibrary.com]

Before applying the CytoNorm method, we characterized the marker distributions of the control and validation samples (Fig. 2). While some small aliquot‐specific differences occurred, the main differences were caused by batch effects: the control and validation samples on the same plate had undergone similar changes in distribution compared to the other plates. When comparing a marker's median values of the 10 plates between the control and validation samples, there was an average correlation of 0.92 (± 0.10) for the unstimulated samples and an average correlation of 0.85 (± 0.15) for the stimulated samples. From this observation, we extrapolated that all samples on one barcoded plate had undergone similar changes. Learning these shifts based on one control sample taken along on all the plates should give us sufficient information to correct for the technical variability caused by the batch effects. As demonstrated with CD15 and CD66 in Figure 2, the batch effects can differ between the different markers, indicating that using beads in only a few channels would be insufficient to correct all batch effects.

Another important characteristic of the batch effects is shown in Figure 2: The shifts were dependent on the level of a marker's expression. Often the lines indicating the 25% quantiles followed a different pattern than the lines indicating the 75% quantiles. Therefore, applying a linear transformation to all cells would not remove the batch effect correctly. The proposed method utilizes spline functions, which can model nonlinear patterns across the expression range.

**Figure 3:**
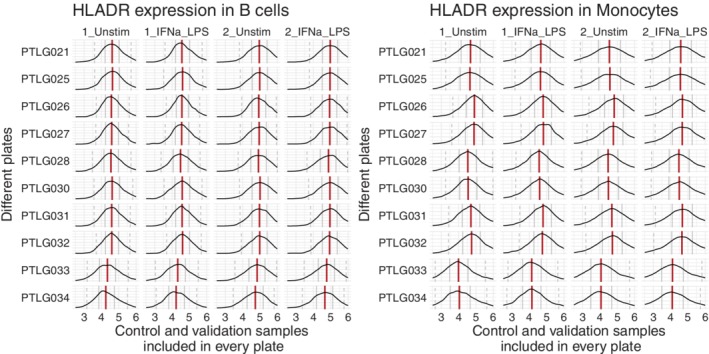
Aliquots of blood samples from two healthy volunteers were measured on 10 separately barcoded plates. We focused on the HLADR intensities for manually gated B cells and monocytes (gated as resp. CD45 + CD66‐CD3‐CD7‐CD11c‐CD123‐ and CD45 + CD66‐CD3‐CD7‐CD11c + CD123‐CD11b + CD33 + CD14+). Even on the same intensity level, these cells underwent different shifts, while similar effects were still occurring between the different samples (note the stronger shift in HLADR signal for PTLG033 and PTLG034 for the monocytes vs. the B cells). The correlations between the median expression values per cell type were resp. 0.99 and 0.97, while across the two cell types, these correlations dropped to 0.79 and 0.86. This indicated that the batch effects were cell‐type specific and a one‐dimensional approach would not be sufficient to remove them. [Color figure can be viewed at http://wileyonlinelibrary.com]

In addition, we noted that some technical variations affected different cell types at different rates. An example is shown in Figure 3, where cells from two distinct populations had similar expression values for the HLA‐DR marker, but showed different patterns depending on other markers (gated as B cells [CD45 + CD66‐CD3‐CD7‐CD11c‐CD123‐] or monocytes [CD45 + CD66‐CD3‐CD7‐CD11c + CD123‐CD11b + CD33 + CD14+]). To handle these cell‐type specific batch effects, we applied a rough clustering on the cytometry data to detect the main cell populations before normalization.

### FlowSOM Metaclusters Are Minimally Effected by Batch Effects

**Figure 4:**
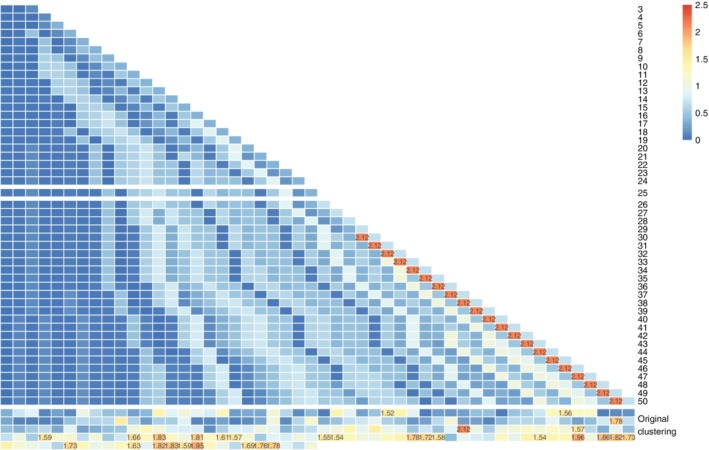
Coefficient of variation results for several clustering results (varied from 3 to 50 final FlowSOM clusters [each one row in the figure] and the 225 original clusters [the bottom 5 rows]). Values are shown on the figure if they are higher than 1.5. We note that as the number of clusters increases, the coefficient of variation increases, as some clusters become batch‐specific. [Color figure can be viewed at http://wileyonlinelibrary.com]

One strong assumption made by the CytoNorm pipeline is that the clusters which are normalized separately, are themselves not affected by the batch effects. We evaluated the proposal of using 25 final clusters on a FlowSOM result based on a 15 × 15 grid. Maximum CV between replicates was 0.87, indicating that on this level, little to no batch effects were influencing the results. We tested multiple numbers of final clusters (going from 3 to 50), and computed the coefficient of variation for the percentage of each of the control samples assigned to the cluster, as shown in Figure 4. Once CV values higher than 2 occur (which is the case for 30 or more clusters), it is likely that some of the clusters are severely impacted by the batch effects, which renders the clustering step inappropriate as a preprocessing step before quantile normalization.

### CytoNorm Aligns Populations between Validation Controls

**Figure 5:**
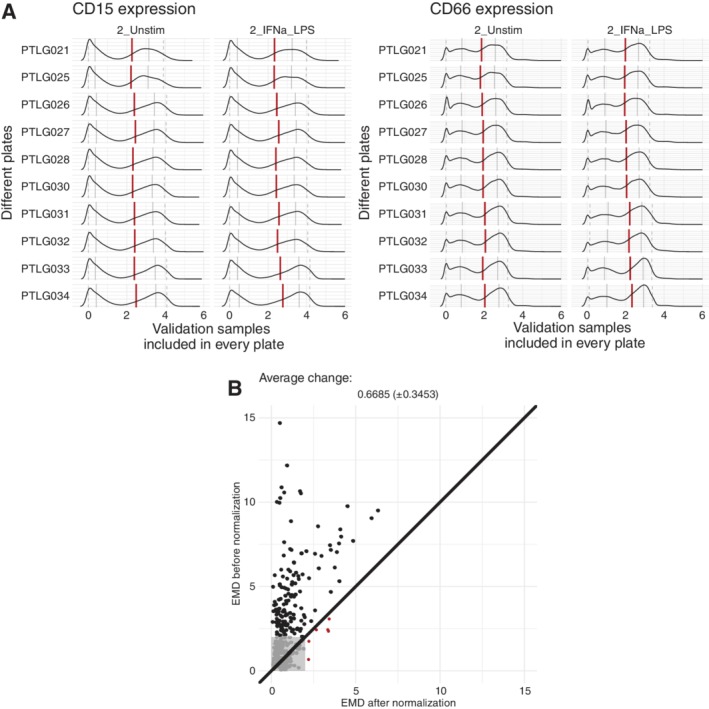
(**A**) Overview of the density distributions of the validation samples after normalization (trained on the control samples). The distributions align much better than before, indicating the removal of the batch effects. (**B**) Comparison of the original distances between unstimulated samples measured for Volunteer 2, versus the distances after normalization using the batch effect model of Volunteer 1. Every dot represents one specific population‐marker combination (36 markers × 10 manually gated cell types = 360 dots). The thick black line indicates the diagonal, all values above this line have decreased after normalization. Dots situated more to the left indicate better results. Red dots indicate values with a bigger distance after normalization than before. Values in the grey area had distances smaller than two before and after normalization, indicating they were not affected by the batch effects and were ignored in the evaluation. For those values affected by the batch effects, normalization with our proposed method resulted in an average decrease of 0.6623 for the unstimulated samples from Volunteer 2, trained on the unstimulated samples from Volunteer 1. [Color figure can be viewed at http://wileyonlinelibrary.com]

We applied the CytoNorm method to normalize the signal variability across the samples. We used either the unstimulated or the stimulated samples of one of the volunteers to learn the batch effects and applied the resulting model to the corresponding samples from the other volunteer. We modeled the batch effects using a FlowSOM grid with 10 × 10 nodes and 25 final clusters, and modeled the splines using 101 quantiles. The new distribution of the data is shown in Figure 5A, analogous to Figure 2 before normalization. Additionally, we computed the EMD values for every marker per cell type, using a manual gating of the data set (see Supporting Information Fig. S1). We applied this measurement on both the original and the normalized samples and plotted those EMD values against each other. A representative result of one experiment for our proposed method is shown in Figure 5B, while Supporting Information Figure S5 gives the full overview of the different samples and methods we tested. In these figures, points on the black diagonal line have the same EMD value before and after normalization. The points above the black line have smaller distances after normalization, while the points below the diagonal have an increased EMD. We conclude that most distances decreased, indicating a reduction in overall batch effect. The average percentage of decrease in distance over the four different testing setups was 0.61 (± 0.39), not taking distances smaller than two into account. In all four of the setups, the EMDs significantly decreased after normalization.

We compared our proposed method with three alternatives: quantile normalization without clustering and approximate min‐max normalization with or without clustering. We compared the results from the approaches with clustering to the results without, as shown in Table 1. When we computed the reduction compared to the original distances, the quantile normalization algorithm resulted in a reduction of only 0.21 (± 1.62). Approximate min‐max normalization applied on clustered cells resulted in a reduction of 0.21 (± 0.62), while approximate min‐max normalization without clustering had a negative average score. Plots comparing the EMDs before and after normalization for all methods are given in Supporting Information Figure S2.

**Table 1:** Overview of the average reduction of all different methods, over all experiments and per individual experiment setup (V1,V2: volunteer used for training, stim,unstim: stimulation of samples used)

	101 quantiles (0, 0.01, 0.02, …, 1)		2 quantiles (0.001, 0.999)	
	With clustering “CytoNorm”	Without clustering	With clustering	Without clustering
Overall score	0.62 (±0.43)	0.21 (±1.62)	0.21 (±0.62)	−0.12 (±0.74)
V1, unstim	0.65 (±0.34)	0.20 (±0.80)	0.26 (±0.46)	−0.10 (±0.69)
V1, stim	0.58 (±0.48)	0.16 (±0.71)	0.21 (±0.58)	−0.30 (±1.04)
V2, unstim	0.69 (±0.41)	0.36 (±0.66)	0.22 (±0.38)	0.05 (±0.47)
V2, stim	0.56 (±0.46)	0.13 (±2.94)	0.15 (±0.90)	−0.09 (±0.57)

### CytoNorm Allows Better Alignment in Dimensionality Reduction Figures

**Figure 6:**
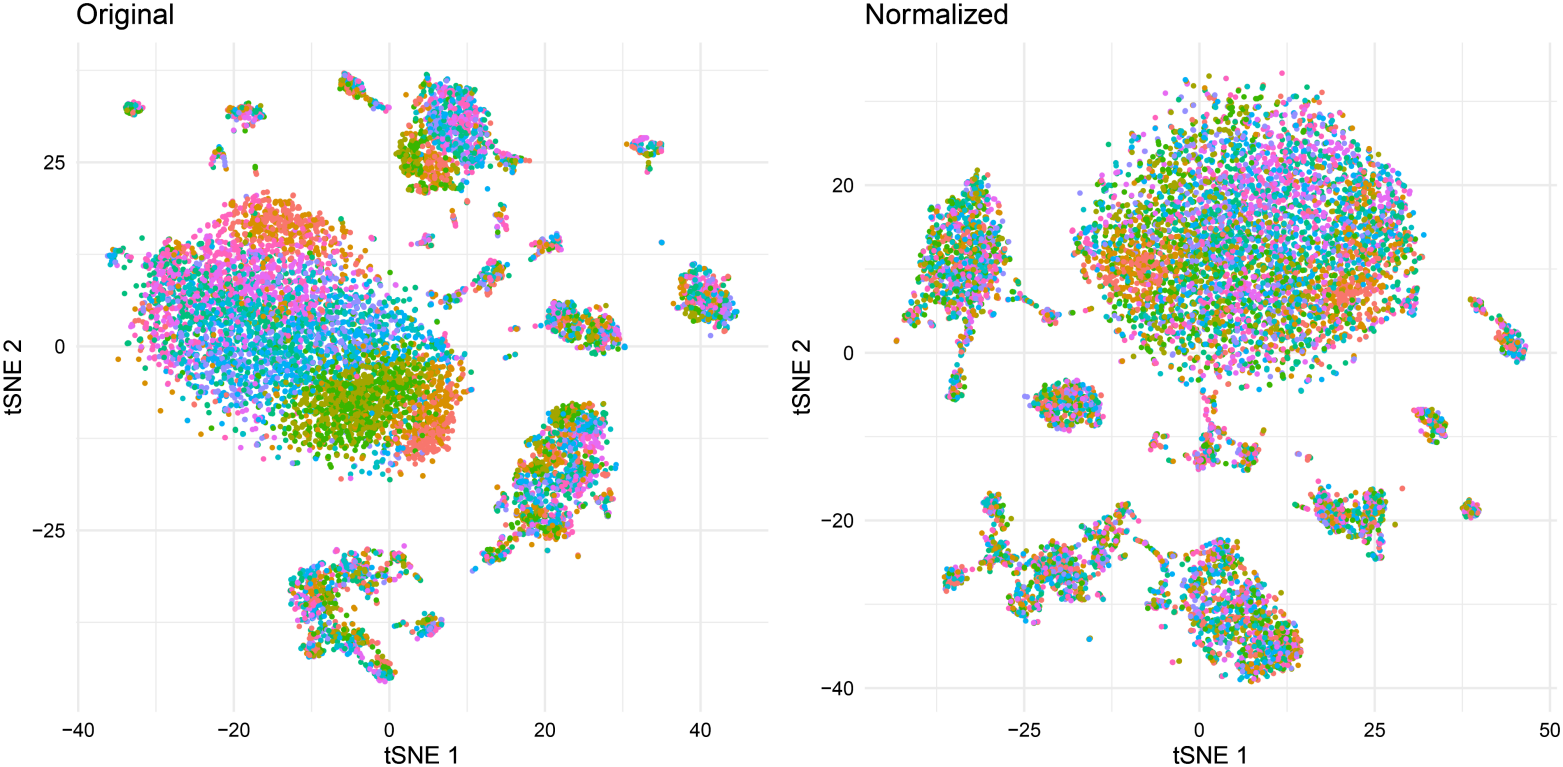
tSNE dimensionality reduction of 10.000 cells sampled from the 10 validation samples (1,000 cells each). Colored by sample (= batch). [Color figure can be viewed at http://wileyonlinelibrary.com]

To have another look at the data, we applied tSNE dimensionality reduction (perplexity 30) on 10.000 cells sampled from the 10 unstimulated validation samples, either of the original data or the data after normalization. When using the original validation samples as input for a tSNE dimensionality reduction, we noticed a batch effect where the location of the cells was impacted by their measurement plate. After normalization, the populations were better aligned, and fewer batch specific regions could be identified (Fig. 6)

### Control Samples Should Span the Full Data Range

It is important that the control samples encompass the whole range of expression values. To demonstrate this, we combined the unstimulated control samples with the stimulated control samples. The cells in these stimulated samples contained intracellular signaling responses that were not present in the unstimulated samples.

**Figure 7:**
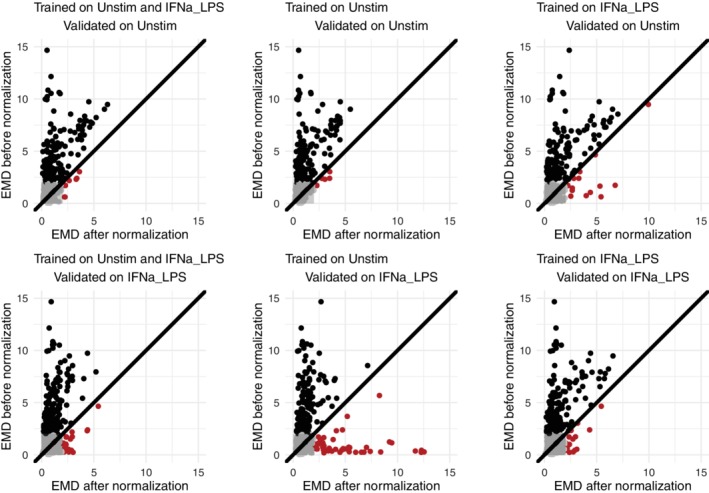
Effect of different stimulations between the training and normalization data set. When the necessary range was not present in the reference samples, extrapolation of the splines introduced wrong transformations, indicated by dots below the diagonal line. [Color figure can be viewed at http://wileyonlinelibrary.com]

We explored three different settings: training on an aggregate of stimulated and unstimulated control samples, training on only the unstimulated control samples or training on only the stimulated control samples. The results on the unstimulated and stimulated validation samples are shown in Figure 7.

When we trained only on the unstimulated sample from Volunteer 1 (middle column), we estimated the batch effects correctly for the unstimulated samples of Volunteer 2: Almost all distances became smaller (top row, middle), as shown in the previous section. However, when we used this model on the stimulated samples, many distances became larger instead of smaller (bottom row, middle, contains many dots under the diagonal in the figure, colored in red). This confirmed that the batch effects were not linear over the range of the expression values. As we could only train on cells where the expression values were low, the model had no information about the batch effects for high expression and made mistakes when extrapolating (example of such a spline given in Supporting Information Fig. S3).

When we trained on the stimulated samples (right column), this problem was resolved and the stimulated samples were correctly normalized (bottom row, right). However, in this case, the unstimulated samples caused a problem because the negative populations were not always represented well in the stimulated samples (top row, right).

The optimal solution was to make sure that the training samples included the whole marker expression range, which was the case when the model was built using both stimulated and unstimulated controls (left column). This model worked well for both the stimulated and unstimulated samples of Volunteer 2 and few red dots appear in either the top or the bottom plot.

### Improved Results for Static Gatings on Clinical Data Set

**Figure 8:**
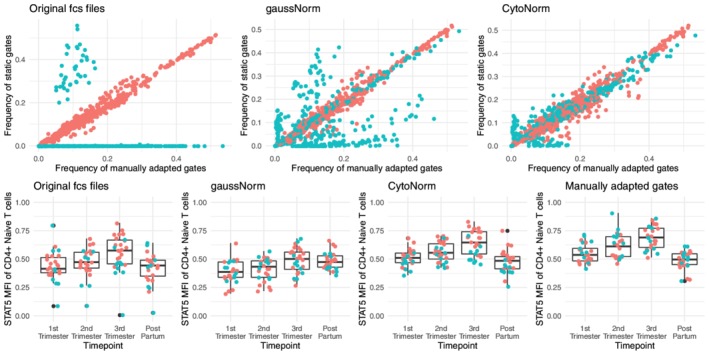
Results on the patient files from the pregnancy study. Red dots: values from original cohort, Blue dots: values from the validation cohort. Top row: comparison of population frequencies acquired by manually adapted gates (x‐axis) and static gating (y‐axis). Applied on files before or after normalization (gaussNorm/CytoNorm). Bottom row: STAT5 MFI values for statically gated CD4+ Naïve T cells. [Color figure can be viewed at http://wileyonlinelibrary.com]

Finally, we applied CytoNorm on the 27 unstimulated patient samples of the pregnancy study (both the original and the validation cohort). The model was trained on the unstimulated control samples, with a FlowSOM grid of 15 by 15 and a final number of 5 clusters (as we saw some increased CV values for 10 clusters, Supporting Information Fig. S4). The goal quantiles were set to be those of the first control sample, rather than the average overall. We then normalized all unstimulated patient samples, and applied a static gating (defined on this first control sample) to all the samples. We compared these static gating results to the manually adapted gating results as reported in the original publication. Results are shown in Figure 8A. It is clear that the validation cohort (blue dots) underwent a big batch effect, strongly skewing the static gating on the original files. While gaussNorm improved the samples, CytoNorm gained results closest to the frequency of the manually adapted gates (closest to the diagonal). When we checked the main parameter that was of relevance in the original paper, we could confirm that the normalization procedure helps to align the data points from the two cohorts better, whereas gaussNorm seemed to have over‐normalized and removed some of the actual biological variation between the samples.

## Discussion

We developed and evaluated CytoNorm, a new normalization method for cytometry data applicable to large clinical studies. We demonstrate that CytoNorm allows reducing mass cytometry signal variability across multiple batches of barcoded samples. However, the algorithm is not limited to mass cytometry data, but could also be applied to other data sets that contain multiple batches, if a reference sample was used and subset‐specific effects are suspected. Notably, CytoNorm could be applied to fluorescence flow cytometry data sets, because many of the causes of technical variance will be the same as for mass cytometry data (e.g., sample preparation happens in a similar manner).

The main requirement for a successful application is that the individual sample variation should be limited compared to the batch‐to‐batch variation. We proposed the CV of the cluster percentages to validate this requirement. It is also important that the reference samples span the full expression range of the samples of interest and consist of cell types that are similar to the ones in the samples of interest. To ensure that the full expression range is captured, it might be necessary to include multiple control samples (e.g., with different stimulations), which can be synthetically combined to learn the batch effects.

To evaluate the strength of the normalization procedure in new settings, it is highly recommended to take two control samples along. Only this way a correct estimation of the impact of the model can be made. In cases where the changes to the control samples are not corresponding to the changes in the other samples, the model might incorrectly transform the data. If taking two controls along with all the batches would not be feasible, it might be useful to at least run a preliminary study with two control samples to test if the same conclusions hold. In this case, it would be recommended to have one control sample from an easily accessible source, which can be taken along on all other batches as the references, and one which is as close to the real samples of interest as possible (same tissue, stimulation, etc.) to test whether the control sample is sufficient to estimate the batch effects on the real samples.

A limitation of our pipeline is the assumption that the batch effects are small enough that the clustering result is minimally impacted. In experiments where large batch effects occur, biologically similar cells might be split in different clusters, which will thus not be aligned. Future research could optimize the clustering step included in the algorithm to avoid these issues. Additionally, it might be difficult to ensure that the control samples span the same expression range as the samples of interest. Future research could optimize the splines to not deviate too far from the identity function in case of extrapolation to avoid spurious artefacts.

In summary, we proposed a normalization strategy for batch effect removal that enables mass cytometry analysis of large clinical cohorts. Importantly, to avoid accidental removal of biologically relevant signals, the algorithm makes no assumptions about the distributions of the clinical samples and relies on the consistency of cellular barcoding and control samples. Also, the algorithm uses a multilayer learning strategy to account for cell‐type specific technical variations. The results demonstrated significant improvements in the quality of primary clinical samples, by reducing the technical variability between the samples.

## Supporting information


**MIFlowCyt**: MIFlowCyt‐Compliant Items

**Supplementary Table 1** Antibody Panel
**Supplementary Figure 1**: Manual gating of the dataset
**Supplementary Figure 2**: Evaluation of multiple normalization algorithms
**Supplementary Figure 3**: Issues with extrapolation
Example of a spline learned on small values only (A) or with few small values (B). x‐values shows the control quantiles, y‐values show the goal quantiles. The red line shows the fitted spline, grey line shows identity function. Both are normalization splines for STAT5 (different clusters, A trained on unstimulated, B trained on stimulated).
**Supplementary Figure 4**: CV values for the unstimulated controls over the original and validation cohort. Tested for 5, 10, 15 or 20 metaclusters and 225 original clusters.
