# Supplementary material for: CytoNorm: A Normalization Algorithm for Cytometry Data
Source: Cytometry A. 2019 Oct 21;97(3):268–78. doi: 10.1002/cyto.a.23904 (PMC7078957; doi:10.1002/cyto.a.23904)
Supplement: Supplementary file 1 — MIFlowCyt: MIFlowCyt‐Compliant Items [file CYTO-97-268-s001.doc]

**Cytometry Part A**

**Author Checklist: MIFlowCyt-Compliant Items**

| **Requirement** | **Please Include Requested Information** |
| --- | --- |
| 1.1. Purpose | Controls included in the studies “An immune clock of human pregnancy” and “Deep Immune Profiling of an Arginine-Enriched Nutritional Intervention in Patients Undergoing Surgery” by Aghaeepour et al. Used in this manuscript to study and correct the batch effects between barcoded plates. |
| 1.2. Keywords | Normalization, Mass cytometry, Whole blood samples, barcoding, healthy controls |
| 1.3. Experiment variables | Untreated,  Stimulated with IFNa and LPS |
| 1.4. Organization name and address | Stanford University School of Medicine  Baxter Laboratory for Stem Cell Biology  269 Campus Drive, CCSR 4215, Stanford CA 94305 |
| 1.5. Primary contact name and email address | Brice Gaudilliere gbrice@stanford.edu |
| 1.6. Date or time period of experiment | Pregnancy dataset: December 2015  Nutritional dataset: August 19, 2013 - June 3, 2015 |
| 1.7. Conclusions | The batch effects between barcoded plates are marker dependent, expression level dependent and cell-type dependent. CytoNorm can remove most of the batch effects, using a reference sample to learn the shifts from. |
| 1.8. Quality control measures |  |
| 2.1.1.1. (2.1.2.1., 2.1.3.1.) Sample description | Whole blood samples from healthy volunteers |
| 2.1.1.2. Biological sample source description | Whole blood |
| 2.1.1.3. Biological sample source organism description | Human |
| 2.1.2.2. Environmental sample location |  |
| 2.3. Sample treatment description | Untreated or stimulated with IFNa and LPS |
| 2.4. Fluorescence reagent(s) description |  |
| 3.1. Instrument manufacturer | Fluidigm |
| 3.2. Instrument model | CyTOF 2.0 mass cytometer |
| 3.3. Instrument configuration and settings |  |
| 4.1. List-mode data files | *We recommend all authors to submit their data files to [http://flowrepository.org](http://flowrepository.org/) and to make them available for the peer-review process. If you have done so, please let us know by inserting the following codes (replace the red text):  1) The link for peer-review process:  [https://flowrepository.org/id/ RvFrBemo4yZYFhRbAOJgXZVnQzHB9uxMeJxbUrC1d3ndbdgv7HCkdZj9zBTnDKy8](https://flowrepository.org/id/%0BRvFrBemo4yZYFhRbAOJgXZVnQzHB9uxMeJxbUrC1d3ndbdgv7HCkdZj9zBTnDKy8).  [https://flowrepository.org/id/ RvFrd1wD1YkixIXSMlfa6Sbb34fVwNNyfK1FUIShv75KEro7gLRorALhRkWMoc7t](https://flowrepository.org/id/%0BRvFrd1wD1YkixIXSMlfa6Sbb34fVwNNyfK1FUIShv75KEro7gLRorALhRkWMoc7t).  [https://flowrepository.org/id/ RvFr9sFH9ejN4sKZnl8ReQwVAvxj4lJzA0MGzjGuEnKUwplECSvKolb4KvcU80I5](https://flowrepository.org/id/%0BRvFr9sFH9ejN4sKZnl8ReQwVAvxj4lJzA0MGzjGuEnKUwplECSvKolb4KvcU80I5).  2) The repository identifier:  <http://flowrepository.org/id/FR-FCM-Z247>  <http://flowrepository.org/id/FR-FCM-Z246>  <http://flowrepository.org/id/FR-FCM-Z248>  This link will be made publicly accessible after the paper is published. |
| 4.2. Compensation description |  |
| 4.3. Data transformation details | Arcsinh transformation with cofactor 5 |
| 4.4.1. Gate description | 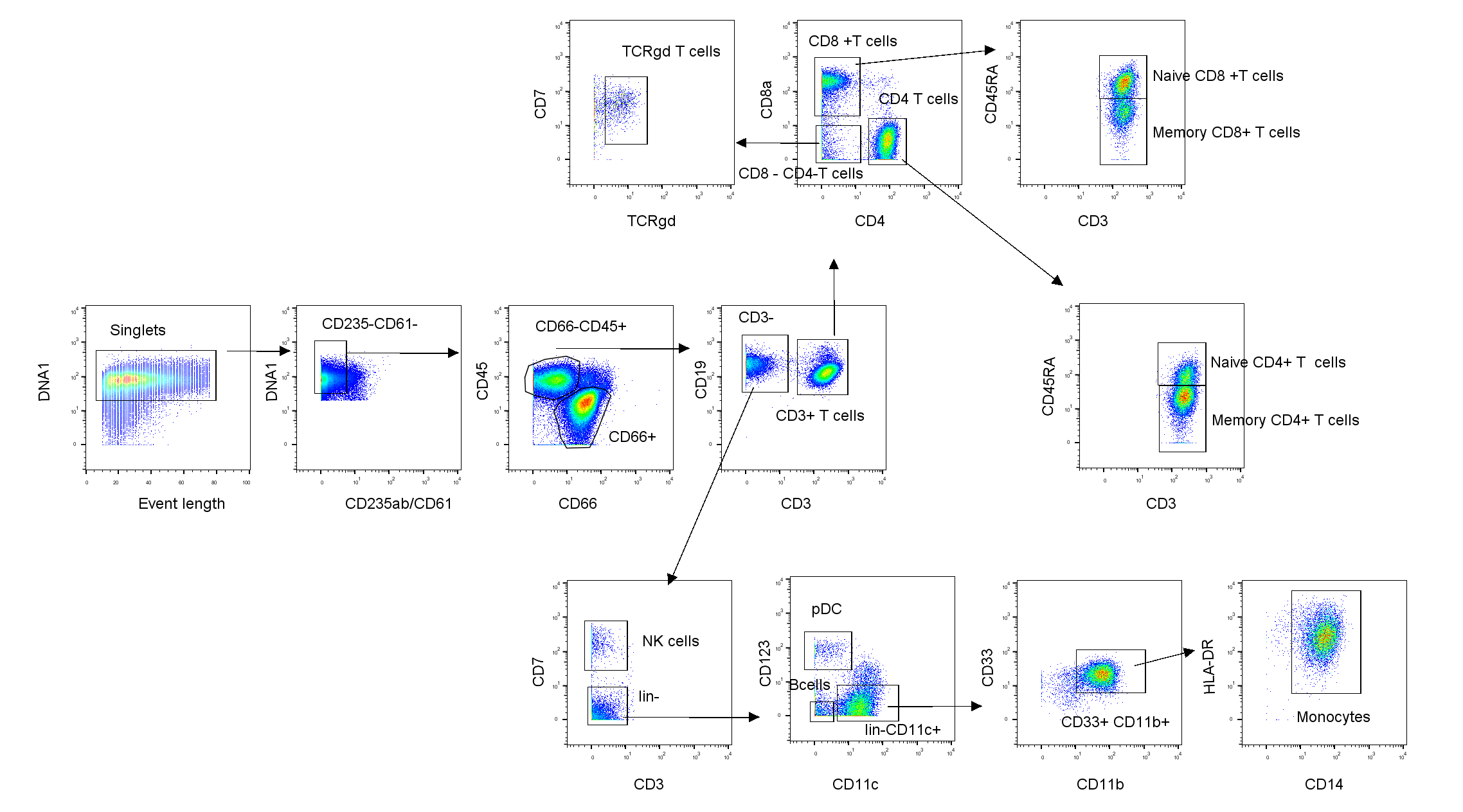 |
| 4.4.2. Gate statistics |  |
| 4.4.3. Gate boundaries |  |

**Notes**

Feel free to use more space than allocated.

You can embed graphics/figures in this document, if needed.

Please make sure to save the document in Microsoft Word version 2003 or older, before uploading to ScholarOne Manuscripts. When uploading this checklist to ScholarOne Manuscripts, please choose the “Supplementary Material for Review” category.

Please note that if your paper is accepted, the checklist will be published as an Online Supporting Information.

For any questions, please contact the Cytometry Part A editorial office at [Cytometrya@wiley.com](mailto:Cytometrya@wiley.com).
