# Supplementary material for: CytoNorm: A Normalization Algorithm for Cytometry Data
Source: Cytometry A. 2019 Oct 21;97(3):268–78. doi: 10.1002/cyto.a.23904 (PMC7078957; doi:10.1002/cyto.a.23904)
Supplement: Supplementary file 2 — Supplementary Table 1 Antibody Panel Supplementary Figure 1: Manual gating of the dataset Supplementary Figure 2: Evaluation of multiple normalization algorithms Supplementary Figure 3: Issues with extrapolation Example of a spline learned on small values only (A) or with few small values (B). x‐values shows the control quantiles, y‐values show the goal quantiles. The red line shows the fitted spline, grey line shows identity function. Both are normalization splines for STAT5 (different clusters, A trained on unstimulated, B trained on stimulated). Supplementary Figure 4: CV values for the unstimulated controls over the original and validation cohort. Tested for 5, 10, 15 or 20 metaclusters and 225 original clusters. [file CYTO-97-268-s002.docx]

## Supplementary Information CytoNorm

Supplementary Table 1: Antibody Panel

Supplementary Figure 1: Manual gating of the dataset


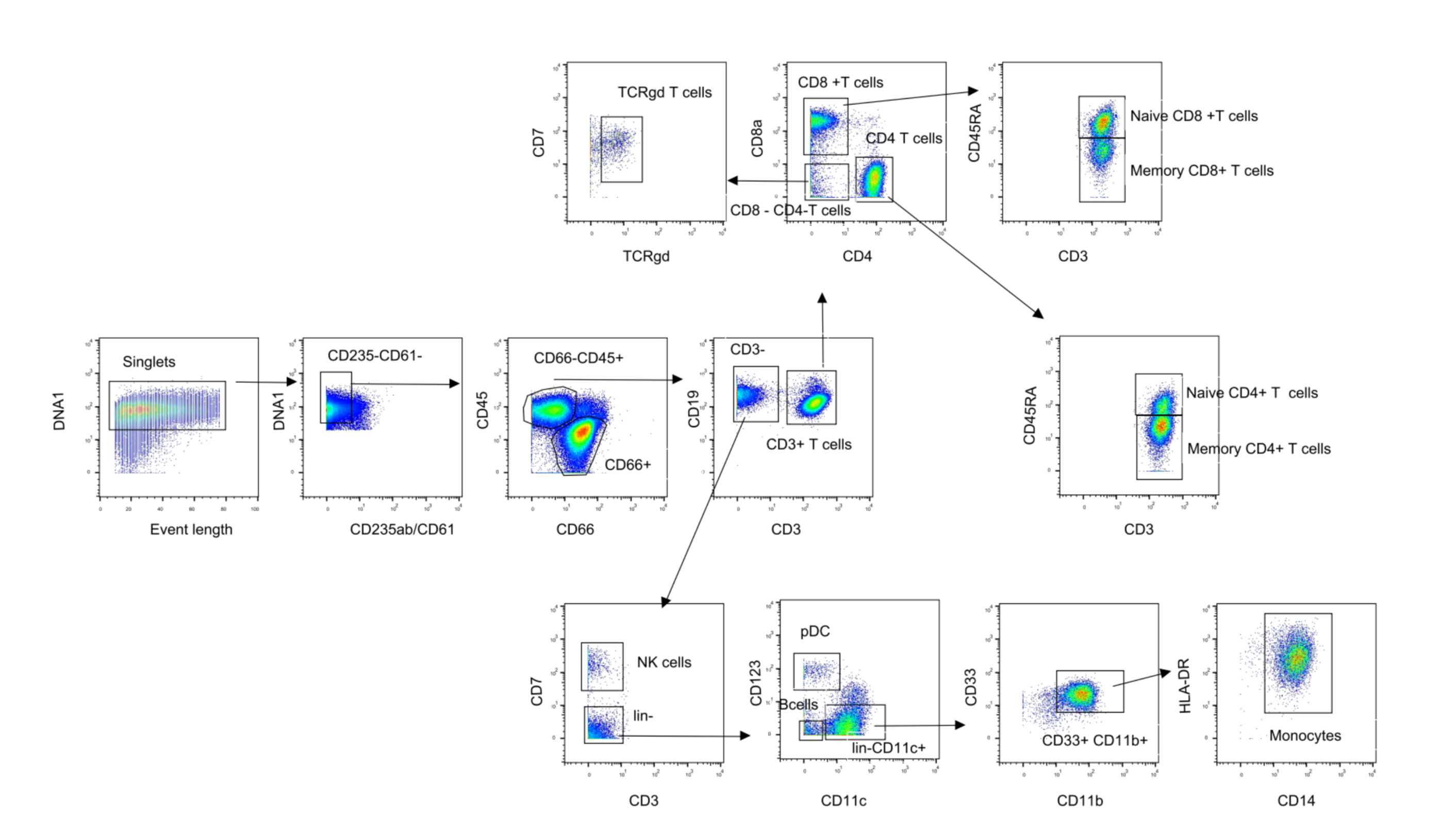


Supplementary Figure 2: Evaluation of multiple normalization algorithms

Supplementary Figure 3: Issues with extrapolation
Example of a spline learned on small values only (A) or with few small values (B). x-values shows the control quantiles, y-values show the goal quantiles. The red line shows the fitted spline, grey line shows identity function. Both are normalization splines for STAT5 (different clusters, A trained on unstimulated, B trained on stimulated).

A: B:

Supplementary figure 4: CV values for the unstimulated controls over the original and validation cohort. Tested for 5, 10, 15 or 20 metaclusters and 225 original clusters.
